# A Time-Symmetric and Retrocausal Resolution of the EPR Paradox

**DOI:** 10.3390/e28030319

**Published:** 2026-03-12

**Authors:** Michael B. Heaney

**Affiliations:** Independent Researcher, 3182 Stelling Drive, Palo Alto, CA 94303, USA; mheaney@alum.mit.edu

**Keywords:** EPR, time-symmetric, retrocausal, paradox

## Abstract

The Copenhagen Interpretation of quantum mechanics explains the Einstein, Podolsky, and Rosen (EPR) experiments with “spooky action at a distance” and nonlocal wavefunction collapse. A time-symmetric and retrocausal interpretation of quantum mechanics explains the same experiments without spooky action at a distance or nonlocal wavefunction collapse. An experiment that can distinguish between the Copenhagen and Time-Symmetric Interpretations is described.

## 1. Introduction

One of the most serious problems facing modern physics is to reconcile special relativity and quantum mechanics [1]. These two foundational theories come into direct conflict in the Einstein, Podolsky, and Rosen (EPR) paradox [2]: “the problem of a genuine Lorentz invariance of our most basic physical theory, namely quantum mechanics, in the face of EPR-Bell experiments is probably the biggest problem that theoretical physics faces today, even though it is not even recognized as such by most theoretical physicists… How does one account for the collapse rule of quantum mechanics in relativistic terms when dealing with entangled states whose parts are spacelike separated? [3]”.

There have been many attempts to explain the EPR paradox using time-symmetric theories [4,5,6,7,8,9,10,11,12,13,14,15]. These papers and books attempt to explain how the spin wavefunctions behave to match the quantum predictions and avoid nonlocality, while ignoring the nonlocal collapse of the spatial wavefunctions. To the best of my knowledge, this paper is the first to explain both the evolution of the spin wavefunctions and the evolution of the spatial wavefunctions without invoking nonlocal wavefunction collapse.

## 2. The Gedankenexperiment

**Figure 1:**
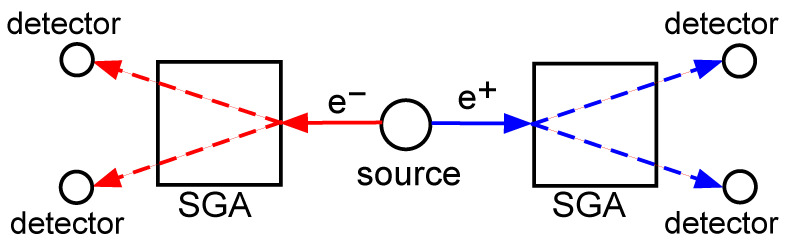
A diagram of the gedankenexperiment. A neutral spin-0 pion at rest in the source decays into a spin-$\frac{1}{2}$ electron and a spin-$\frac{1}{2}$ positron, which then travel in opposite directions. Each particle enters a Stern–Gerlach apparatus (SGA) capable of separating spin-up ${|↑\rangle }_{z}$ and spin-down ${|↓\rangle }_{z}$ particles. The two particles are subsequently detected in one of the detectors on the left side and one of the detectors on the right side. The red arrows show the possible paths of the electron, while the blue arrows show the possible paths of the positron.

Freedman and Clauser [16] carried out the first experimental proof of nonlocal quantum entanglement in 1972. Figure 1 shows a simplified version of their experiment. A source at the center of the experiment emits neutral spin-0 pions at rest with respect to the apparatus. Each pion decays into an electron and a positron, which move in opposite directions towards the two Stern–Gerlach apparatuses. The Stern–Gerlach apparatuses separate each particle into either spin-up or spin-down, which are then detected by one of the two detectors following each Stern–Gerlach apparatus. This experiment did not close the locality loophole. The locality loophole is when the detectors on either side of the experiment can communicate with each other by a luminal or subluminal signal. This allows the possibility of the detectors on either side of the experiment to coordinate their measurement settings to mimic nonlocal action at a distance.

Aspect et al. [17,18] carried out the next significant experiments on nonlocal quantum entanglement in 1982. These experiments confirmed the violation of Bell’s inequality to a higher degree of certainty, but did not close the locality loophole.

Zeilinger et al. [19] carried out further significant experiments on nonlocal quantum entanglement in 1998. These experiments eliminated the locality loophole by separating the detections on the left and right sides by a spacelike interval, so it was not possible for the electron and positron to communicate with each other upon detection. Zeilinger et al. [20] also experimentally demonstrated quantum teleportation in 1997, a key component of quantum computation networks.

Hensen et al. [21] closed the nonlocality and fair sampling loopholes simultaneously in 2015. The fair sampling loophole is the assumption that the detected particles are representative of all the particles, detected and undetected.

## 3. The Copenhagen Interpretation (CI) Explanation

**Figure 2:**
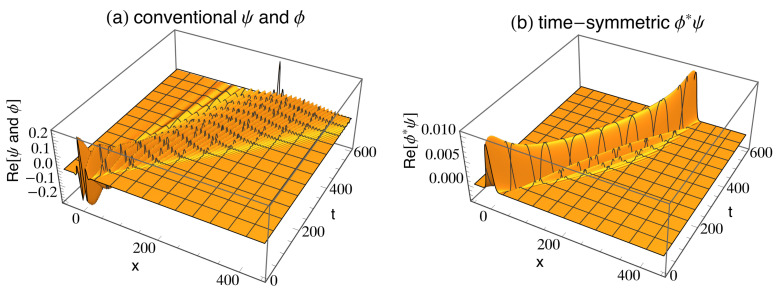
(**a**) The real parts of the Copenhagen spatial traveling Gaussian wavefunctions ψ(x,t) and ϕ(x,t). The wavefunction ψ(x,t) evolves in spacetime from being centered at $\left(x_{i},t_{i}\right)=\left(0,0\right)$ until collapsing to ϕ(x,t) at $\left(x_{f},t_{f}\right)=\left(250,600\right)$. (**b**) The real part of the time-symmetric spatial transition amplitude density $\phi^{*}\left(x,t\right)\psi \left(x,t\right)$. The transition amplitude density evolves continuously in spacetime from being centered at $\left(x_{i},t_{i}\right)=\left(0,0\right)$ to being centered at $\left(x_{f},t_{f}\right)=\left(250,600\right)$. There is no nonlocal collapse. The imaginary parts of the wavefunctions and transition amplitude density give little new information, and are not shown. These figures do not show the zitterbewegung effect.

The CI assumes that a retarded spatial wavefunction that satisfies the Schrödinger equation and evolves forwards in time from initial conditions until collapsing upon measurement gives the most complete description of a particle that is in principle possible [22]. For example, the retarded traveling Gaussian spatial wavefunction in one dimension

(1) $$\psi \left(x,t\right)=\frac{1}{\sqrt{\pi }}{\left(\frac{1}{\sigma +i(t-t_{i})/\sigma }\right)}^{1/2}exp\left[\frac{-{\left(x-x_{i}\right)}^{2}}{4\sigma^{2}+2i\left(t-t_{i}\right)}\right]exp\left[i\frac{k\left(x-x_{i}\right)-k^{2}\left(t-t_{i}\right)/2}{1+i\left(t-t_{i}\right)/\left(2\sigma^{2}\right)}\right],$$

where *x* is the location of the particle, $\left(x_{i},t_{i}\right)=\left(0,0\right)$ are the emission location and time, all particle masses are set to 1, the initial gaussian width σ=5, the momentum k=0.5, and natural units are used: ℏ=c=1. Figure 2a shows the evolution of this retarded spatial wavefunction, which collapses upon measurement by a detector to the retarded spatial wavefunction ϕ(x,t) at $\left(x_{f},t_{f}\right)=\left(250,600\right)$, where

(2) $$\phi \left(x,t\right)=\frac{1}{\sqrt{\pi }}{\left(\frac{1}{\sigma +i(t-t_{f})/\sigma }\right)}^{1/2}exp\left[\frac{-{\left(x-x_{f}\right)}^{2}}{4\sigma^{2}+2i\left(t-t_{f}\right)}\right]exp\left[i\frac{k\left(x-x_{f}\right)-k^{2}\left(t-t_{f}\right)/2}{1+i\left(t-t_{f}\right)/\left(2\sigma^{2}\right)}\right].$$

Now consider the spin part of the retarded wavefunction. The CI assumes the two particles are created with the entangled spin singlet wavefunction

(3) $$|spin\rangle =(|↑↓\rangle -\left|↓↑\rangle \right)/\sqrt{2},$$

where the red arrows refer to the electron, the blue arrows refer to the positron, and the spatial wavefunction is suppressed for clarity. The total quantum state is simply the tensor product of Equations (1) and (3). There are no subscripts specifying axes because this is a rotationally invariant wavefunction. When the two SGA’s $\hat{z}$-axes are parallel to each other, every run results in the detection of a spin-up particle on one side and a spin-down particle on the other side. When one or the other particle is detected, the CI assumes the spin singlet wavefunction collapses randomly to either $|{↑↓\rangle }_{z}$ or $|{↓↑\rangle }_{z}$, collapsing the other particle to the opposite sign of spin, even when a spacelike interval separates the two particles. When the two SGA’s $\hat{z}$-axes are at an angle θ to each other, the probability of perfect anticorrelation varies as $cos^{2}\left(\theta /2\right)$, while the probability of perfect correlation varies as $sin^{2}\left(\theta /2\right)$. This agrees with the measured experimental results.

## 4. The Time-Symmetric Interpretation (TSI) Explanation

Time-symmetric explanations of quantum behavior predate the discovery of the Schrödinger equation. Time-symmetric interpretations of quantum mechanics have been carefully justified and checked for over a century [23], and have recently been extended to mixed states [24].

In 1922 Tetrode [25] published a paper describing a time-symmetric treatment of electrons interacting via light radiation. He wrote: “…for the moment we would like to treat the positive and negative direction of time as equal. It then follows that we must take the arithmetic mean of the retarded and advanced potentials. We then obtain for the four-potential Φ in well-known notation

(4) $$\Phi =\frac{1}{2}\int {\left[P\right]}_{t}-\frac{r}{c}\frac{dS}{r}+\frac{1}{2}\int {\left[P\right]}_{t}+\frac{r}{c}\frac{dS}{r},$$

where *P* is the four-current… Although according to the classical point of view radiation is simply emitted at random, only to be possibly absorbed somewhere else, according to our theory, however, emission and absorption are processes that require each other, and with each emission it is already predetermined when, where and how the absorption is to be taken place… The sun would not radiate if it were alone in the cosmos without any other bodies that could absorb its radiation. In our theory there is no fundamental distinction between heat radiation and heat conductance, and just as impossible it is for a body to lose heat through conduction when the molecules of its surface are not in interaction with those of the other body, just as impossible it is for this through radiation, only the distances are much greater in the last case.”

In 1955 Watanabe [26] developed the Double Inferential Vector Formalism (DIVF), where the product of a retarded wavefunction from the source and an advanced wavefunction from the sink fully described quantum transitions. In 2001 Aharonov et al. [27] rediscovered and extended the DIVF, renaming it the Two State Vector Formalism (TSVF). Wavefunction collapse is a necessary part of the TSVF.

The next significant step was the development of the Transactional Interpretation (TI) [28] by Cramer in 1986. He proposed every quantum transition requires a retarded wavefunction from a source and an advanced wavefunction from a sink, with the two wavefunctions added together. He used the TI to explain the Freedman-Clauser experiment, Renninger’s null result gedankenexperiment, Wheeler’s delayed choice experiment, Schrödinger’s cat, Wigner’s friend, the Hanbury-Brown–Twiss interferometer, and the Albert–Aharonov–D’Amato predictions.

The Time-Symmetric Interpretation (TSI) [29] used in this paper was developed in 2013. The TSI assumes that a transition amplitude density (TAD) gives the most complete description of a quantum system that is in principle possible. This TAD is the algebraic product of two wavefunctions: a retarded wavefunction $\psi (\vec{r},t)$ that obeys the Copenhagen Schrödinger equation and satisfies the initial boundary conditions, and an advanced wavefunction $\phi^{*}\left(\vec{r},t\right)$ that obeys the complex conjugate of the Schrödinger equation and satisfies the final boundary conditions [30]. I speculate that the retarded and advanced wavefunctions are both emitted stochastically, and a transition occurs when an advanced wave from a sink arrives at the source at the same time as a retarded wave is leaving the source. This could solve the quantum measurement problem. It is assumed that these two wavefunctions never collapse nonlocally. We can then define the TAD as the ontology of the TSI. The TAD looks like a localized wavepacket traveling from the source to the sink, as shown in Figure 2b. The TAD diverges from the source and then converges to the sink, without nonlocal collapse. The emission and absorption mechanisms are assumed to occur at the TAD termini.

In 1932, Dirac showed that all of the experimental predictions of the Copenhagen Interpretation can be formulated in terms of transition probabilities [31]. The Time-Symmetric Interpretation inverts this fact by postulating that quantum mechanics is a theory which predicts transition probabilities. This implies the Time-Symmetric Interpretation has the same validity as the Copenhagen Interpretation. For example, the advanced traveling Gaussian wavefunction is

(5) $$\phi^{*}\left(x,t\right)=\frac{1}{\sqrt{\pi }}{\left(\frac{1}{\sigma -i(t-t_{f})/\sigma }\right)}^{1/2}exp\left[\frac{-{\left(x-x_{f}\right)}^{2}}{4\sigma^{2}-2i\left(t-t_{f}\right)}\right]exp\left[-i\frac{k\left(x-x_{f}\right)-k^{2}\left(t-t_{f}\right)/2}{1-i\left(t-t_{f}\right)/\left(2\sigma^{2}\right)}\right],$$

where *x* is the location of the particle, $\left(x_{f},t_{f}\right)=\left(250,600\right)$ is the detection location and time, the final Gaussian width is σ=5, the momentum k=0.5, all particle masses are set to 1, and natural units are used: ℏ=c=1. The probability of a transition is given by $P_{s}=A_{s}^{*}A_{s}$, where

(6) $$A_{s}\equiv \int_{-\infty }^{\infty }dx\phi^{*}\left(x,t\right)\psi \left(x,t\right).$$

All relativistic wave equations have both retarded and advanced solutions, and both types of solutions survive in the nonrelativistic limit. One consequence of the assumptions of the TSI is that a source will not emit a quantum unless there is a sink somewhere that can absorb the quantum. This is supported by the experimental confirmation of the Purcell effect [32].

Let us now describe the spin part of the retarded and advanced wavefunctions. Consider first the case where the two SGA’s $\hat{z}$-axes are parallel to each other. The source will emit either $|{↑↓\rangle }_{z}$ or $|{↓↑\rangle }_{z}$ particle pairs. These particle pairs are not entangled because in the TSI only indistinguishable transition amplitude densities are entangled [30]. The particle pair spins are antiparallel because angular momentum is conserved. The source emits particles polarized along the $\hat{z}$ axes because the advanced waves from the four detectors, after passing through the two SGAs, will be polarized along the $\hat{z}$ axes. The final states of the detectors have a retrocausal influence on the initial orientation of the electron and positron spins. This may be a kind of mutual causation that works in both time directions symmetrically. Assume the source emits a $|{↑↓\rangle }_{z}$ [$|{↓↑\rangle }_{z}$] particle pair. The only nonzero TADs occur when the upper [lower] left detector emits a $\langle {↑|}_{z}$ [$\langle {↓|}_{z}$] advanced wave and the lower [upper] right detector emits a $\langle {↓|}_{z}$ [$\langle {↑|}_{z}$] advanced wave. The possible TADs are then $\langle {↑↓|↑↓\rangle }_{z}$ [$\langle {↓↑|↓↑\rangle }_{z}$]. These give perfect anticorrelations, in agreement with the experimental results.

Now consider the case where the two SGA’s $\hat{z}$-axes are at an angle θ to each other. Let’s assume the left SGA is oriented along the $\hat{z}$-axis while the right SGA is oriented along the $\hat{n}$-axis. The source will have to emit either $|{↑↓\rangle }_{z}$, $|{↓↑\rangle }_{z}$, $|{↑↓\rangle }_{n}$, or $|{↓↑\rangle }_{n}$ particle pairs to conserve angular momentum and retrocausally match some of the advanced waves from the detectors. This again may be a kind of mutual causation that works in both time directions symmetrically. Consider the case where the source emits a $|{↑↓\rangle }_{z}$ [$|{↓↑\rangle }_{z}$] particle pair. The advanced waves from the upper [lower] left detector, after passing through the $\hat{z}$ SGA, will produce only $\langle {↑|}_{z}$ [$\langle {↓|}_{z}$] advanced waves at the source, while the advanced waves from the lower [upper] right detector, after passing through the $\hat{n}$ SGA, will produce only $\langle {↓|}_{n}$ [$\langle {↑|}_{n}$] advanced waves at the source. For the $|{↑↓\rangle }_{z}$ [$|{↓↑\rangle }_{z}$] particle pair, one particle (say the $|{↑\rangle }_{z}$ [$|{↓\rangle }_{z}$]) will go to the $\hat{z}$ SGA with amplitude 1, and the other particle will go to the $\hat{n}$ SGA with amplitude 〈↓|↓〉z[nn〈↑|↑〉z]=cos(θ/2). The difference in angular momentum could be taken up by the SGA. The same argument holds for the $|{↑↓\rangle }_{n}$ and $|{↓↑\rangle }_{n}$ particle pairs produced by the source. The probability of perfect anticorrelation then varies as $cos^{2}\left(\theta /2\right)$, while the probability of perfect correlation varies as $sin^{2}\left(\theta /2\right)$. This also agrees with the measured experimental results.

## 5. Discussion

Einstein defined “local causality” as causal influences traveling continuously through spacetime at or below the speed of light [33]. The Copenhagen Interpretation explanation of the EPR paradox violates local causality: the measurement of the electron spin as $|{↑\rangle }_{z}$ at one detector causes the positron spin at the other detector to collapse to $|{↓\rangle }_{z}$ at a spacelike interval. This is spooky action at a distance. It is often claimed that special relativity forbids retrocausal signals. But this is not true: it is the combination of special relativity and the principle of macroscopic local causality that forbids retrocausal signals. The principle of macroscopic local causality is equivalent to the macroscopic entropic arrow of time. But quantum phenomena often happen at a microscopic level and can violate the second law of thermodynamics, for example in fluctuation phenomena. This allows the possibility of microscopic influences traveling backwards in time, provided they do not transfer classical signals [34]. This also allows the possibility of advanced wavefunctions in quantum mechanics, which travel backwards in time but do not transfer classical signals.

How far back in time can advanced wavefunctions travel? Tetrode suggested advanced photons can travel from Earth to stars many light years away [25]. Photons are a special case, since they have zero rest mass and energies much higher than the effective energy of room temperature. For more massive particles, the limit may be set by the coherence length/time of macroscopic quantum phenomena.

The time-symmetric and retrocausal theory in this paper is local in the sense that there is no action at a distance. It also shows how nonlocal wavefunction collapse, which is in direct conflict with special relativity, is not necessary to explain quantum phenomena.

There is an experiment that can distinguish between the Copenhagen Interpretation (CI) and the Time-Symmetric Interpretation (TSI) [29]. To understand this, we need to start with the relativistic versions of these two theories. The CI postulates that a quantum particle is described by a wavefunction $\Theta (\vec{r},t)$ and initial conditions. The CI also postulates that the wavefunction $\Theta (\vec{r},t)$ of a free spin-0 particle of mass *m* evolves according to the relativistic Klein-Gordon equation (KGE):

(7) $$\left(\frac{1}{c^{2}}\frac{\partial^{2}}{\partial t^{2}}-\nabla^{2}+\frac{m^{2}c^{2}}{\hbar^{2}}\right)\Theta =0.$$

The KGE has both positive energy $E_{+}$ and negative energy $E_{-}$ solutions, with energies:

(8) $$E_{\pm }=\pm \sqrt{p^{2}c^{2}+m^{2}c^{4}}.$$

The CI assumes the general solution $\Theta (\vec{r},t)$ is a sum of a retarded wavefunction $\Psi (\vec{r},t)$ and an advanced wavefunction $\Phi^{*}\left(\vec{r},t\right)$:

(9) $$\Theta =\Psi +\Phi^{*}.$$

The complex conjugate of the KGE is:

(10) $$\left(\frac{1}{c^{2}}\frac{\partial^{2}}{\partial t^{2}}-\nabla^{2}+\frac{m^{2}c^{2}}{\hbar^{2}}\right)\Theta^{*}=0.$$

We can multiply Equation (7) on the left by $\Theta^{*}$, multiply Equation (10) on the left by Θ, then take the difference and rearrange terms to get:

(11) $$\frac{\partial }{\partial t}\left[\frac{i\hbar }{2mc^{2}}\left(\Theta^{*}\frac{\partial \Theta }{\partial t}-\Theta \frac{\partial \Theta^{*}}{\partial t}\right)\right]+\nabla \cdot \left[\frac{\hbar }{2mi}\left(\Theta^{*}\nabla \Theta -\Theta \nabla \Theta^{*}\right)\right]=0.$$

We will define $\rho (\vec{r},t)$ as:

(12) $$\rho \equiv \frac{i\hbar }{2mc^{2}}\left(\Theta^{*}\frac{\partial \Theta }{\partial t}-\Theta \frac{\partial \Theta^{*}}{\partial t}\right),$$

and $\vec{j}\left(\vec{r},t\right)$ as

(13) $$\vec{j}\equiv \frac{\hbar }{2mi}\left(\Theta^{*}\nabla \Theta -\Theta \nabla \Theta^{*}\right),$$

to get the local conservation law

(14) $$\frac{\partial \rho }{\partial t}+\nabla \cdot \vec{j}=0.$$

For the CI general solution to the KGE given by Equation (9), the CI probability density $\rho (\vec{r},t)$ given by Equation (12) will contain terms that oscillate at a frequency $\omega \approx 2mc^{2}/\hbar$, due to interference between the positive and negative energy terms. This same behavior occurs for the general solution to the Dirac equation for an electron, where $\omega \approx 10^{21}\mathrm{Hz}$. Schrödinger discovered the possibility of this in 1930, naming it zitterbewegung [35].

In the TSI, a complete experiment is described by a transition amplitude composed of two wavefunctions: a retarded wavefunction $\Psi (\vec{r},t)$, which satisfies the initial conditions; and an advanced wavefunction $\Phi^{*}\left(\vec{r},t\right)$, whose complex conjugate $\Phi (\vec{r},t)$ satisfies the final conditions.

Let us propose TSI evolution postulates for a free, spin-0 particle of mass *m*, which say the retarded wavefunction $\Psi (\vec{r},t)$ evolves from initial conditions according to the KGE:

(15) $$\left(\frac{1}{c^{2}}\frac{\partial^{2}}{\partial t^{2}}-\nabla^{2}+\frac{m^{2}c^{2}}{\hbar^{2}}\right)\Psi =0,$$

while the advanced wavefunction $\Phi^{*}\left(\vec{r},t\right)$ evolves from final conditions according to the complex conjugate of the KGE

(16) $$\left(\frac{1}{c^{2}}\frac{\partial^{2}}{\partial t^{2}}-\nabla^{2}+\frac{m^{2}c^{2}}{\hbar^{2}}\right)\Phi^{*}=0.$$

If we multiply Equation (15) on the left by $\Phi^{*}$, multiply Equation (16) on the left by Ψ, take the difference of the two resulting equations, and rearrange terms, we get:

(17) $$\frac{\partial }{\partial t}\left[\frac{i\hbar }{2mc^{2}}\left(\Phi^{*}\frac{\partial \Psi }{\partial t}-\Psi \frac{\partial \Phi^{*}}{\partial t}\right)\right]+\nabla \cdot \left[\frac{\hbar }{2mi}\left(\Phi^{*}\nabla \Psi -\Psi \nabla \Phi^{*}\right)\right]=0.$$

Now we will define $\rho_{s}\left(\vec{r},t\right)$ as:

(18) $$\rho_{s}\equiv \frac{i\hbar }{2mc^{2}}\left(\Phi^{*}\frac{\partial \Psi }{\partial t}-\Psi \frac{\partial \Phi^{*}}{\partial t}\right),$$

and define $\vec{j_{s}}\left(\vec{r},t\right)$ as

(19) $$\vec{j_{s}}\equiv \frac{\hbar }{2mi}\left(\Phi^{*}\nabla \Psi -\Psi \nabla \Phi^{*}\right),$$

to get a TSI local conservation law

(20) $$\frac{\partial \rho_{s}}{\partial t}+\nabla \cdot \vec{j_{s}}=0.$$

In the TSI, a free particle is described by the TSI amplitude density $\rho_{s}\left(\vec{r},t\right)$ given by Equation (18), where $\Psi (\vec{r},t)$ is a positive energy wavefunction and $\Phi^{*}\left(\vec{r},t\right)$ is a negative energy wavefunction. The two zitterbewegung terms cancel each other out in $\rho_{s}\left(\vec{r},t\right)$ and $\vec{j_{s}}\left(\vec{r},t\right)$. This means the position of a free spin-0 particle will never display zitterbewegung. Measuring zitterbewegung is not currently possible but may be possible with further technological advances.

## 6. Conclusions

The Copenhagen Interpretation explains the EPR paradox by invoking nonlocal action at a distance and nonlocal wavefunction collapse. The Time-Symmetric Interpretation explains the same paradox without invoking nonlocal action at a distance or nonlocal wavefunction collapse. This resolves the ninety-year-old conflict between quantum mechanics and special relativity.

The Copenhagen Interpretation predicts that an electron at rest in free space will oscillate at a frequency of $\omega \approx 10^{21}$ Hz, while the Time-Symmetric Interpretation predicts no oscillation. This frequency is too high to measure with current technology but may be possible in the future. This experiment would distinguish between these two interpretations.

## Data Availability

No new data were created or analyzed in this study.
